# Supplementary material for: Pyruvate Dehydrogenase Kinase 1 inhibition mediated oxidative phosphorylation enhancement in cartilage promotes osteoarthritis progression
Source: BMC Musculoskelet Disord. 2023 Jul 20;24:597. doi: 10.1186/s12891-023-06585-6 (PMC10357736; doi:10.1186/s12891-023-06585-6)
Supplement: Supplementary file 1 — Additional file 1. [file 12891_2023_6585_MOESM1_ESM.zip › BP.Gsea.1653623667859/GOBP_MITOCHONDRIAL_FUSION.html]

Details for gene set GOBP\_MITOCHONDRIAL\_FUSION[GSEA]

|  || Dataset | 55457\_collapsed\_to\_symbols.cls.cls #OA\_versus\_normal.cls.cls #OA\_versus\_normal\_repos |
| Phenotype | cls.cls#OA\_versus\_normal\_repos |
| Upregulated in class | OA |
| GeneSet | GOBP\_MITOCHONDRIAL\_FUSION |
| Enrichment Score (ES) | 0.5051399 |
| Normalized Enrichment Score (NES) | 1.6888281 |
| Nominal p-value | 0.004761905 |
| FDR q-value | 0.1671522 |
| FWER p-Value | 1.0 |
Table: GSEA Results Summary

  

Fig 1: Enrichment plot: GOBP\_MITOCHONDRIAL\_FUSION      
 Profile of the Running ES Score & Positions of GeneSet Members on the Rank Ordered List

  

| SYMBOL | TITLE | RANK IN GENE LIST | RANK METRIC SCORE | RUNNING ES | CORE ENRICHMENT || 1 | PARL | presenilin associated rhomboid like [Source:HGNC Symbol;Acc:HGNC:18253] | 14 | 1.156 | 0.1583 | Yes |
| 2 | FIS1 | "fission, mitochondrial 1 [Source:HGNC Symbol;Acc:HGNC:21689]" | 250 | 0.785 | 0.2484 | Yes |
| 3 | ZDHHC6 | zinc finger DHHC-type palmitoyltransferase 6 [Source:HGNC Symbol;Acc:HGNC:19160] | 837 | 0.559 | 0.2800 | Yes |
| 4 | MFF | mitochondrial fission factor [Source:HGNC Symbol;Acc:HGNC:24858] | 932 | 0.531 | 0.3460 | Yes |
| 5 | MIEF1 | mitochondrial elongation factor 1 [Source:HGNC Symbol;Acc:HGNC:25979] | 1531 | 0.423 | 0.3579 | Yes |
| 6 | MUL1 | mitochondrial E3 ubiquitin protein ligase 1 [Source:HGNC Symbol;Acc:HGNC:25762] | 1610 | 0.412 | 0.4087 | Yes |
| 7 | STOML2 | stomatin like 2 [Source:HGNC Symbol;Acc:HGNC:14559] | 1819 | 0.382 | 0.4452 | Yes |
| 8 | PRKN | parkin RBR E3 ubiquitin protein ligase [Source:HGNC Symbol;Acc:HGNC:8607] | 1972 | 0.364 | 0.4835 | Yes |
| 9 | CHCHD3 | coiled-coil-helix-coiled-coil-helix domain containing 3 [Source:HGNC Symbol;Acc:HGNC:21906] | 2279 | 0.329 | 0.5051 | Yes |
| 10 | PID1 | phosphotyrosine interaction domain containing 1 [Source:HGNC Symbol;Acc:HGNC:26084] | 3280 | 0.222 | 0.4581 | No |
| 11 | MTCH2 | mitochondrial carrier 2 [Source:HGNC Symbol;Acc:HGNC:17587] | 4147 | 0.142 | 0.4105 | No |
| 12 | THG1L | tRNA-histidine guanylyltransferase 1 like [Source:HGNC Symbol;Acc:HGNC:26053] | 4417 | 0.120 | 0.4061 | No |
| 13 | VAT1 | vesicle amine transport 1 [Source:HGNC Symbol;Acc:HGNC:16919] | 4779 | 0.088 | 0.3903 | No |
| 14 | BNIP3 | BCL2 interacting protein 3 [Source:HGNC Symbol;Acc:HGNC:1084] | 5501 | 0.032 | 0.3387 | No |
| 15 | HUWE1 | "HECT, UBA and WWE domain containing E3 ubiquitin protein ligase 1 [Source:HGNC Symbol;Acc:HGNC:30892]" | 6558 | -0.046 | 0.2631 | No |
| 16 | MFN2 | mitofusin 2 [Source:HGNC Symbol;Acc:HGNC:16877] | 7064 | -0.085 | 0.2356 | No |
| 17 | GDAP1 | ganglioside induced differentiation associated protein 1 [Source:HGNC Symbol;Acc:HGNC:15968] | 7172 | -0.093 | 0.2402 | No |
| 18 | MFN1 | mitofusin 1 [Source:HGNC Symbol;Acc:HGNC:18262] | 8460 | -0.191 | 0.1666 | No |
| 19 | OPA1 | OPA1 mitochondrial dynamin like GTPase [Source:HGNC Symbol;Acc:HGNC:8140] | 8655 | -0.205 | 0.1797 | No |
| 20 | AFG3L2 | AFG3 like matrix AAA peptidase subunit 2 [Source:HGNC Symbol;Acc:HGNC:315] | 9045 | -0.235 | 0.1820 | No |
| 21 | BAK1 | BCL2 antagonist/killer 1 [Source:HGNC Symbol;Acc:HGNC:949] | 9266 | -0.253 | 0.1998 | No |
| 22 | BAX | "BCL2 associated X, apoptosis regulator [Source:HGNC Symbol;Acc:HGNC:959]" | 9323 | -0.257 | 0.2309 | No |
| 23 | TFRC | transferrin receptor [Source:HGNC Symbol;Acc:HGNC:11763] | 10184 | -0.339 | 0.2109 | No |
Table: GSEA details [plain text format]

  

Fig 2: GOBP\_MITOCHONDRIAL\_FUSION      
 Blue-Pink O' Gram in the Space of the Analyzed GeneSet

  

Fig 3: GOBP\_MITOCHONDRIAL\_FUSION: Random ES distribution      
 Gene set null distribution of ES for **GOBP\_MITOCHONDRIAL\_FUSION**
